# Reaction of Stabilized Criegee Intermediates from Ozonolysis of Limonene with Water: *Ab Initio* and DFT Study

**DOI:** 10.3390/ijms14035784

**Published:** 2013-03-12

**Authors:** Lei Jiang, Ru Lan, Yi-Sheng Xu, Wen-Jie Zhang, Wen Yang

**Affiliations:** 1 State Key Laboratory of Environmental Criteria and Risk Assessment, Chinese Research Academy of Environmental Sciences, Beijing 100012, China; E-Mails: jiangle3657@sina.com (L.J.); zhangwj@craes.org.cn (W.-J.Z.); yangwen@craes.org.cn (W.Y.); 2 College of Environmental Sciences and Engineering, Peking University, Beijing 100871, China; 3 China Waterborne Transport Research Institute, Beijing 100088, China; E-Mail: lanru@wti.ac.cn

**Keywords:** volatile organic chemicals (VOCs), ozone, *ab initio* methods, ozonolysis reaction mechanisms, limonene

## Abstract

The mechanism of the chemical reaction of H_2_O with three stabilized Criegee intermediates (stabCI-OO, stabCI-CH_3_-OO and stabCI*x*-OO) produced via the limonene ozonolysis reaction has been investigated using *ab initio* and DFT (Density Functional Theory) methods. It has been shown that the formation of the hydrogen-bonded complexes is followed by two different reaction pathways, leading to the formation of either OH radicals via water-catalyzed H migration or of α-hydroxy hydroperoxide. Both pathways were found to be essential sources of atmospheric OH radical and H_2_O_2_ making a significant contribution to the formation of secondary aerosols in the Earth’s atmosphere. The activation energies at the CCSD(T)/6-31G(d) + CF level of theory were found to be in the range of 14.70–21.98 kcal mol^−1^. The formation of α-hydroxy hydroperoxide for the reaction of stabCI*x*-OO and H_2_O with the activation energy of 14.70 kcal mol^−1^ is identified as the most favorable pathway.

## 1. Introduction

The stabilized Criegee intermediates (stabilized carbonyl oxides) from the ozonolysis of alkenes can react with various atmospheric compounds [1–5], particularly with the formaldehyde, H_2_O, NO*_x_*, SO_2_, H_2_SO_4_ and CO and many others. One of the most important reactions in the atmospheric chemistry is the reaction of stabilized Criegee intermediates with water, the dominant constituent of condensable vapors in the Earth’s atmosphere. This reaction is considered to be one of the major degradation reactions in the Earth’s atmosphere [6,7]. It is also known that this reaction leads to the formation of α-hydroxy hydroperoxides (HOCH_2_OOH or HMHP), organic acids, ketones, aldehydes, OH radicals, and H_2_O_2_[6,8–19]. These species have been detected in both the ambient air and precipitation, in both forested and urban areas under polluted conditions [15,20–25]. HMHP may act as an enzymatic inhibitor of peroxidases, while H_2_O_2_ is a very important oxidant [26–28]. The H_2_O_2_ contributes to acid precipitation via the conversion of SO_2_ into H_2_SO_4_[29], which is the key atmospheric nucleation precursor. The reaction of stabilized Criegee intermediate with water can be an additional essential source of OH radicals [30]. The oxidation of H_2_O by stabilized Criegee intermediates in ozonolysis of alkenes can also contribute to the formation of secondary organic aerosol (SOA) [31,32]. Jonsson *et al.*[31] corroborated the aforementioned conclusion for the ozonolysis of limonene, Δ^3^-carene, and α-pinene at low concentrations of the aforementioned species and noticed that the particle number and mass concentration increases with increasing relative humidity The reaction of stabilized Criegee intermediate with water is also important for the water purification and waste water processes, where ozonolysis is widely used [33].

**Scheme 1:**
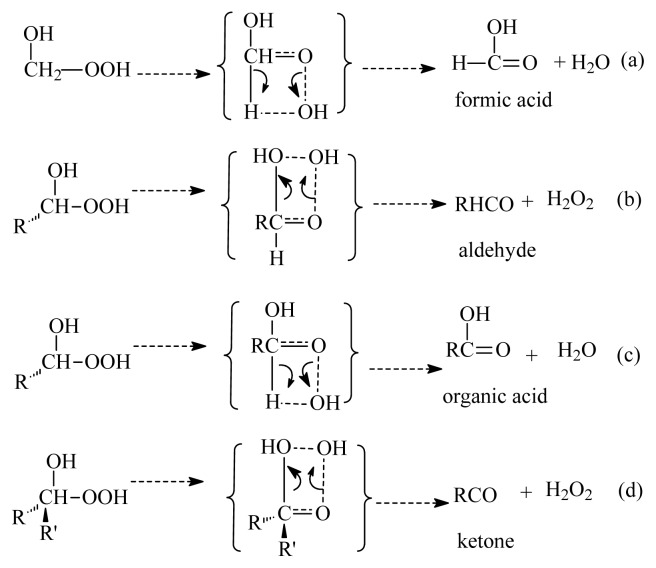
Different fates of RR′C (OH)OOH formed in the reaction of the stabilized Criegee intermediate and water depending on the chemical nature of the R and R′ substitutes.

Previous experimental and theoretical studies on the reaction of H_2_O with CH_2_OO and CH_3_HCOO, (CH_3_)_2_COO and the stabilized Criegee intermediate from ozonolysis of limonene indicate that this reaction is the three-step mechanism [2,6,34–38]. At the first step, a hydrogen-bond complex is formed (a). At the second step, two decomposition pathways for the hydrogen-bond complex are possible: water-catalyzed hydrogen (H) migration would lead to the formation of OH radical via OO bond breaking (b). At the final stage, the α-hydroxy hydroperoxide is formed by the addition of the water molecule to the stabilized Criegee intermediate. Sauer *et al.*[14] found that α-hydroxy hydroperoxides RR′C(OH)OOH formed in the reaction of the stabilized Criegee intermediate and water may have different fates depending on the chemical nature of the R and R′ substitutes (see Scheme 1).

**Figure 1:**
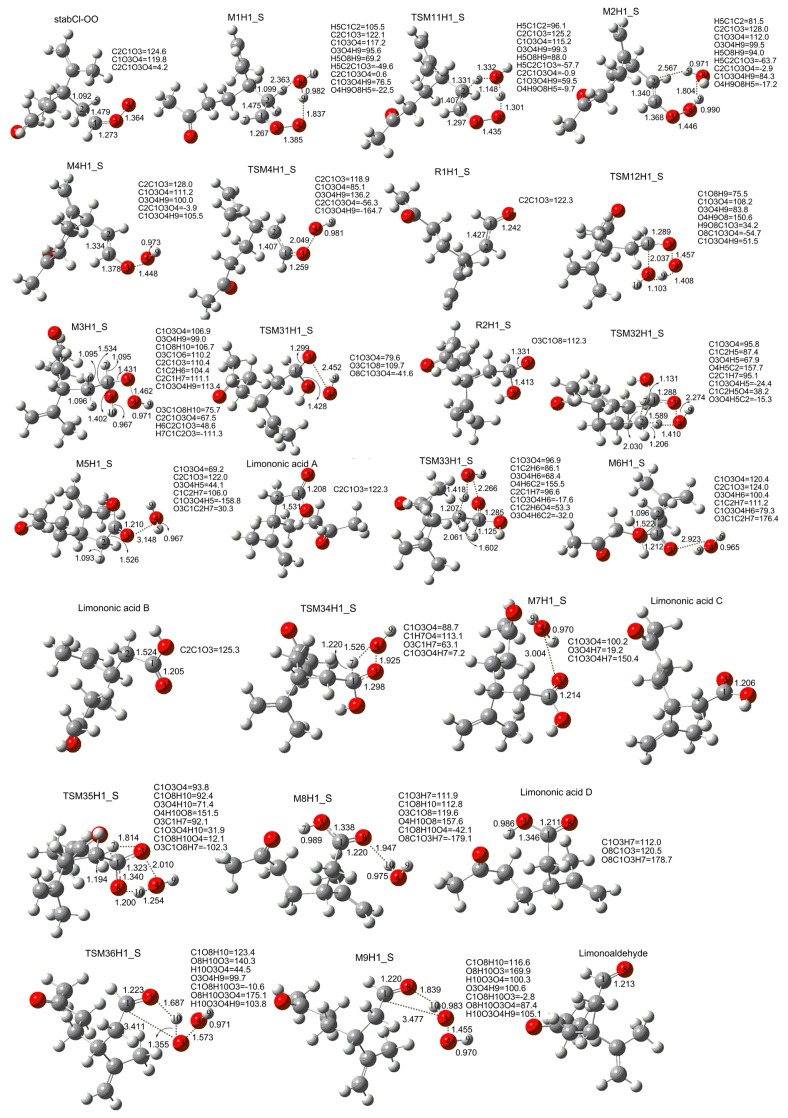
Geometries of the stationary points in the stabCI-OO + H_2_O reaction obtained at the B3LYP/6-31G(d,p) level of theory. Bond lengths and intermolecular distances are given in Å.

**Figure 2:**
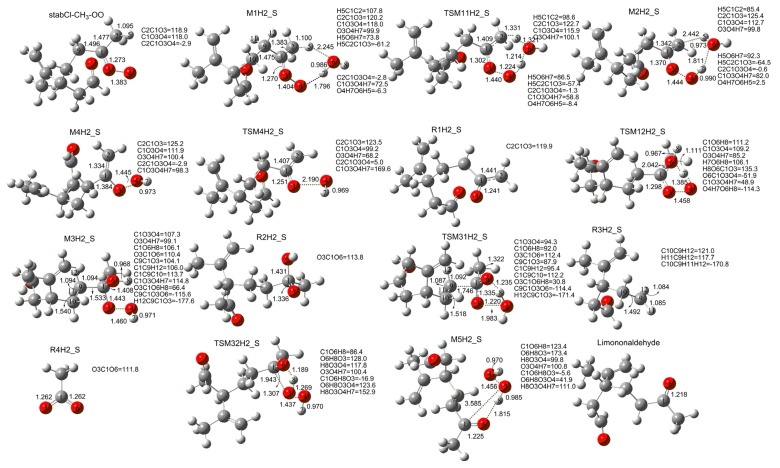
Geometries of the stationary points in the stabCI-CH_3_-OO + H_2_O reaction obtained at the B3LYP/6-31G(d,p) level of theory. Bond lengths and intermolecular distances are given in Å.

**Figure 3:**
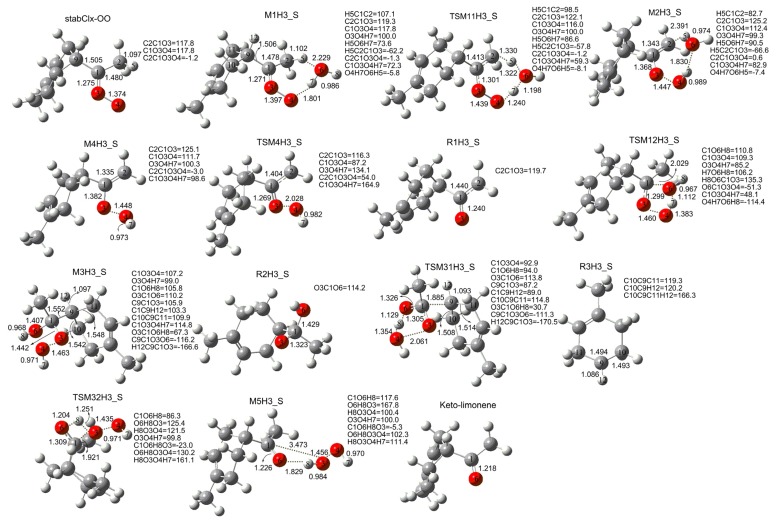
Geometries of the stationary points in the stabCIx-OO + H_2_O reaction obtained at the B3LYP/6-31G(d,p) level of theory. Bond lengths and intermolecular distances are given in Å.

Being produced by more than 300 species, limonene or 4-isopropenyl-1-methyl-cyclohexene, is the most abundant monoterpene in the Earth’s troposphere [39,40]. The reaction of ozone with limonene, which has both endocyclic and exocyclic double bonds, is one of the most important oxidation processes in the Earth’s troposphere. Ozonolysis of limonene is an essential source of OH radicals, H_2_O_2_ and plays a significant role in the formation of atmospheric aerosols via the oxidation of H_2_O by stabilized Criegee intermediate [31]. There are four stabilized Criegee intermediate formed from cleavage of the O_3_-Limonene primary ozonide: stabCI-OO (See stabCI-OO structure in Figure 1) and stabCI-CH_3_-OO (See stabCI-CH_3_-OO structure in Figure 2) formed from the endocyclic primary ozonide decomposition and stabCI*x*-OO (See stabCI*x*–OO structure in Figure 3) and stabCH_2_OO formed from the exocyclic primary ozonide decomposition [41].

**Scheme 2:**
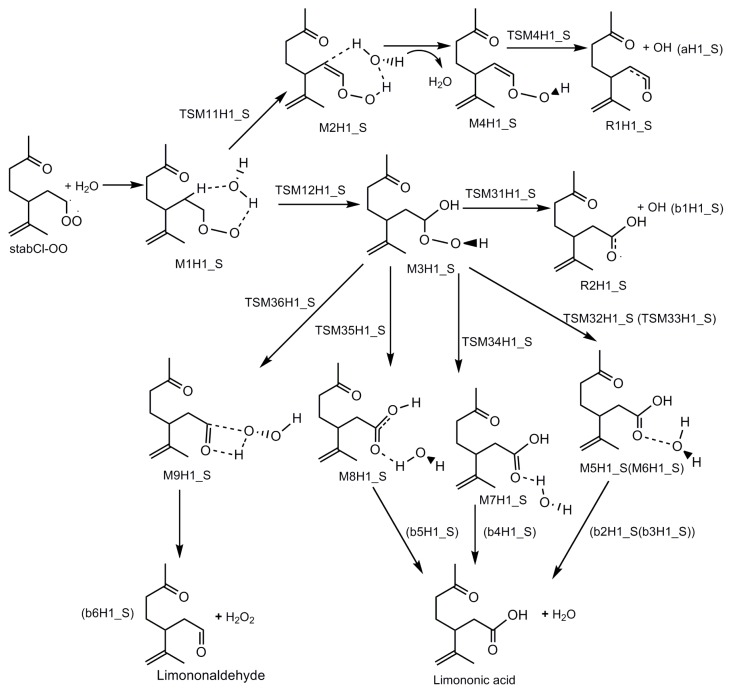
Mechanistic diagram for the reaction between H_2_O and stabCI-OO arising from the limonene ozonolysis.

**Scheme 3:**
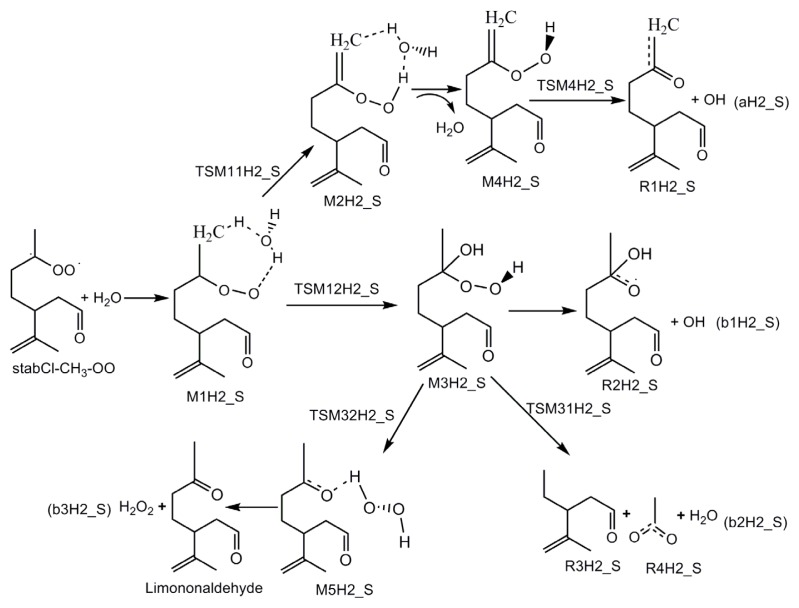
Mechanistic diagram for the reaction between H_2_O and stabCI-CH_3_-OO arising from the limonene ozonolysis.

**Scheme 4:**
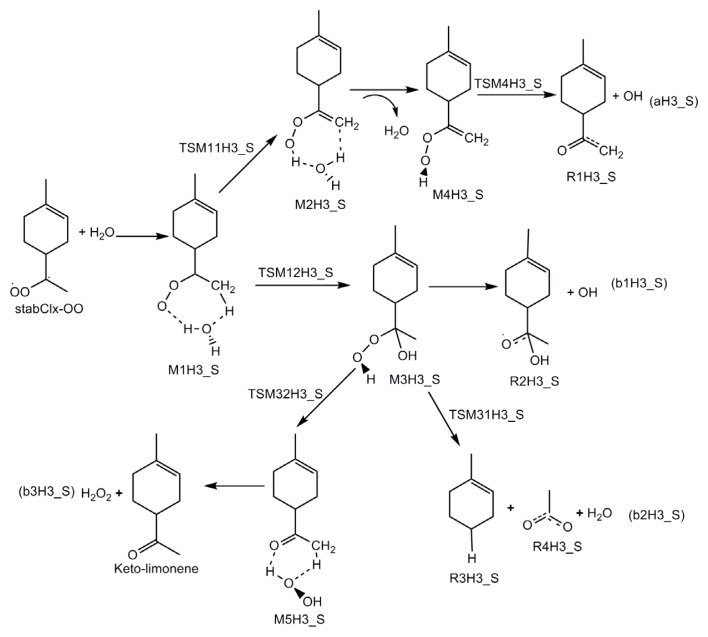
Mechanistic diagram for the reaction between H_2_O and stabCI*x*-OO arising from the limonene ozonolysis.

The mechanistic diagrams for the reactions of H_2_O with three stabilized Criegee intermediates (stabCI-OO, stabCI-CH_3_-OO and stabCI*x*-OO) from ozonolysis of limonene are shown in Schemes 2–4, respectively. The structures of the intermediate adduct and transition states are denoted as M and TS, respectively. The H1_S, H2_S and H3_S are used to distinguish the reactions of H_2_O with stabCI-OO, stabCI-CH_3_-OO and stabCI*x*-OO. The reactions of H_2_O with all the three stabilized Criegee intermediates are three-step reactions. For each reaction, a hydrogen-bond complex is initially formed. The further evolution of the hydrogen-bond complex formed occurs via two different reaction pathways:

1. Formation of OH radicals with water-catalyzed H migration. The water molecule assists the H migration toward the terminal oxygen of the COO moiety, which could lead to the formation of OH radicals.
2. Formation of α-hydroxy hydroperoxide. Cycloaddition of H_2_O to stabilized Criegee intermediate, where the oxygen of water is linked to the carbon atom of the stabilized Criegee intermediate while a hydrogen atom of water is transferred to the terminal oxygen of the COO unit.

The reaction pathway (2) have different fates depending on the nature of the R and R′ substitutes in subsequent reactions. For reaction between stabCl-OO and H_2_O, the M1H1_S (hydrogen-bond complex) is getting transformed via the two main reaction pathways (pathway (1) and pathway (2)) through the corresponding transition states (TSM11H1_S and TSM12H1_S), producing M2H1_S and M3H1_S, respectively. In pathway (1), M2H1_S evolves via losing water molecule and the transition state (TSM4H1_S), to produce OH and R1H1_S radicals (pathway (aH1_S)). M3H1_S in pathway (2) may have different degradation paths. M3H1_S can be transformed via the transition state (TSM31H1_S) into OH and R2H1_S radicals (pathway (b1H1_S)), limononic acid and H_2_O (pathway (b2H1_S-b5H1_S)) and limononaldehyde and H_2_O_2_ (via the transition states (TSM32H1_S-TSM35H1_S) (pathway (b6H1_S)). Moreover, the pathway (b4H1_S) was also found for the reaction between H_2_O and stabCl-OO in the recent study of Wang [42].

In the reaction between stabCI-CH_3_-OO and H_2_O, the hydrogen-bonded complex M1H2_S evolves via their corresponding transition states (TSM11H2_S and TSM12H2_S), leading to the formation of M2H2_S and M3H2_S, respectively. In pathway (1), M2H2_S evolves via the detachment of the water molecule and the transition state (TSM4H2_S), to produce OH and R1H2_S radicals (pathway (aH2_S)). M3H2_S may follow three different reaction pathways in pathway (2). M3H2_S can be transformed into OH and R2H2_S radicals (pathway (b1H2_S)) or via the transition states (TSM31H2_S and TSM32H2_S), into H_2_O, R3H2_S, R4H2_S, limononaldehyde and H_2_O_2_. The reaction between stabCI*x*-OO and H_2_O follows the same pathways as those for the reaction of stabCI-CH_3_-OO and H_2_O. The M1H3_S can evolve via their corresponding transition states (TSM11H3_S and TSM12H3_S) to formation of OH radical, R1H3_S radical, R2H3_S radical, R3H3_S, R4H3_S, H_2_O, Keto-limonene and H_2_O_2_.

The formations of limononic acid, limononaldehyde and keto-limonene are consistent with the experimental results for the reaction between three stabilized Criegee intermediates and H_2_O in Limonene-ozone reaction in UNC smog chamber [32,43].

Although the important role of the reaction between Criegee intermediates from ozonolysis of limonene and H_2_O for the formation of SOA and organic acids affecting both the global climate changes and public health is well established, the mechanism of these reactions remains poorly understood. Since the stability of the Criegee intermediates is moderately weak, the computational quantum chemistry is the most efficient for determining the geometric and electronic structures of these chemically activated complexes. Since reaction between the simple stabCH_2_OO and H_2_O has been investigated in some detail [6,11,44–51] in the past, the mechanism of this reaction is left out of the scope of the present paper.

In the present paper, the reaction of H_2_O with three more complex stabilized Criegee intermediates (stabCI-OO, stabCI-CH_3_-OO and stabCI*x*-OO) from ozonolysis of limonene has been investigated in order to gain new insights of the oxidation mechanisms under atmospheric conditions. *Ab initio* and DFT methods have been employed to obtain geometries and energies of the transition states and subsequent degradation products. Reaction and activation energies for the reaction between the three stabilized Criegee intermediates and H_2_O have been computed at different levels of theory, including the higher level CCSD(T)/6-31G(d) + CF. A thorough thermochemical analysis has been carried out and its results and implications for the ozonolysis of limonene have been discussed.

## 2. Computational Details

The computations were performed using GAUSSIAN 03 suite of programs [52] on the SGI ALTIX 4700 supercomputer. The geometry optimization of all reactants, stabilized Criegee intermediates, transition states, and products was executed using Becke’s three-parameter hybrid method employing the LYP correction function (B3LYP) with the split valence polarized basis set 6-31G(d,p) [10,53]. The stationary points were classified as minima in the case, when no imaginary frequencies were found, and as a transition state in the case, when only one imaginary frequency was obtained. In order to verify the transition states connecting reactants and products, the intrinsic reaction coordinate (IRC) analysis [54] at the B3LYP/6-31G(d,p) level of theory has also been applied to each transition state of every reaction. We just freely optimized the original three CI structure to get the minima conformer, then keeping the conformer for the succedent reactions. (see Figures 1–3). There exists difference between our optimized conformation and those in other studies. For example, in our study, we have chosen opposite orientation on stabCI-OO and stabCI*x*-OO from what Baptista *et al.* did [55]. In general, we think our method of computational process is applicable for explanation of unanimous reaction between the same conformer of CI with water. The DFT structures were then used in the single-point energy calculations using frozen core second-order Møller-Plesset perturbation theory (MP2) and coupled-cluster theory with single and double excitations including perturbative corrections for the triple excitations (CCSD(T) [56] with various basis sets. The basis set effects on calculated energies for the reactions of stabilized Criegee intermediates with H_2_O were corrected at MP2 level according to the developed method, which has been successfully applied for studying the complex reaction mechanisms and pathways of volatile organic compounds (VOCs) in the atmosphere [57]. A correction factor (CF) has been determined from the energy difference between the MP2/6-31G(d) and MP2/6-311++G(d,p) levels. Energies calculated at the CCSD(T)/6-31G(d) level of theory have been corrected using the aforementioned MP2 level corrections. The application of the CF has been validated in several studies of isoprene and limonene reactions initiated by NO_3_, OH and O_3_[57–61]. For example, new important results have been obtained using the same method for the reaction of stabilized Criegee intermediates from the ozonolysis of limonene with sulfur dioxide in the recent paper [41]. These considerations lead us to conclude that the CCSD(T)/6-31G(d) + CF selected as a primary method and calculation scheme are appropriate for studying the reactions of VOCs with ozone or OH radical and description of the Criegee intermediates in the ozonolysis of VOCs reaction [35,37,47,62–66].

## 3. Results and Discussion

### 3.1. Reaction Mechanism

The reactions of H_2_O and limonene stabilized Criegee intermediates (stabCI-OO, stabCI-CH_3_-OO and stabCI*x*-OO) occur via the six main reaction pathways. Each transition state has only one imaginary harmonic vibrational frequency and can be classified as the first-order saddle point. The values of imaginary frequencies for TSM11H1_S, TSM12H1_S, TSM11H2_S, TSM12H2_S, TSM11H3_S and TSM12H3_S transition states are 1516.76i, 434.95i, 1584.16i, 590.54i, 1586.31i and 593.62i, respectively.

Figure 1 presents the optimized geometries of the stationary points for the reaction of stabCI-OO and H_2_O obtained at the B3LYP/6-31G(d,p) level of theory and the most important corresponding geometrical properties such as bond lengths and bond angles. Scheme 2 and Figure 1 show that the reactions of stabCI-OO with H_2_O initially lead to the formation of the hydrogen-bond complex M1H1_S. Then the M1H1_S evolves via the transition state TSM11H1_S (formation of OH radicals with water-catalyzed H migration path or reaction (1)), to the hydrogen-bond complex M2H1_S, which can evolve into hydroperoxide M4H1_S and water. Finally, the cleavage of the O-OH bond in M4H1_S may lead to OH and R1H1_S radicals via the transition state TSM4H1_S along the reaction path (aH1_s).

As seen from Figure 1, there are some major changes in main bonds. In particular, the hydrogen-bond M1H1_S complex includes a seven-membered ring. The distance between O4 and H9 connecting the terminal oxygen atom (O4) of the C1O3O4 group belonging to stabCI-OO to the hydrogen atom (H9), is 1.837 Å, while the length of the O8–H9 bond is 0.982 Å. The length of H5–O8 bond, which connects the oxygen (O8) of original water to the hydrogen atom (H5), is 2.363 Å. The corresponding transition (TSM11H1_S) shows that the transfer of the hydrogen atom (H5) linked to the carbon atom (C2) to the water molecule can take place, and the transfer of one hydrogen atom (H9) from water to the terminal oxygen of the C1O3O4 group can also occur at the same time. This indicates that the water molecule acts as a catalyst of the hydrogen migration. A comparison of the corresponding transition (TSM11H1_S) structure with M1H1_S shows that the C2-H5 bond length increases by 0.232 Å to 1.331 Å, while the H5–O8 distance decreases by 1.031 Å to 1.332 Å. The O8–H9 bond length increases by 0.166 Å to 1.148 Å, while the O4-H9 bond length decreases by 0.536 Å to 1.301 Å. C2–H5 and O8–H9 distances in TSM11H1_S continue to increase, leading to both C2–H5 and O8–H9 bonds broken and the formation of O8–H5 and O4–H9 bonds. At the same time, the hydrogen-bond complex M2H1_S is formed. The lengths of C2–H5, H5–O8, O8–H9 and O4–H9 bonds in M2H1_S are 2.567 Å, 0.971 Å, 1.804 Å, 0.990 Å and 1.446 Å, respectively. A hydroperoxide M4H1_S and water are formed from the M2H1_S. The O3–O4 and O4–H9 bonds in M4H1_S experiencing minor changes during this process are of 1.448Å and 0.973 Å, respectively. Finally, the O–OH bond in M4H1_S is broken to form of O4H9 and R1H1_S radicals via the TSM4H1_S transition state. The O3–O4 bond in the peroxide (2.049 Å) in TSM4H1_S complex gets elongated by 0.601 Å compared to that in the M4H1_S. The reaction (1) may be considered as a possible significant source of atmospheric OH radicals. However, this pathway is a poor OH source. Because the vinyl hydroperoxides (M4H1_S) formed in this pathway is easily to collisionally stabilized under atmospheric condition and thus impeded OH formation [67,68].

M1H1_S can also evolve via the transition state TSM12H1_S (formation of α-hydroxy hydroperoxide or reaction (2)), into a α-hydroxy hydroperoxide M3H1_S. In this case, the formation of transition state TSM12H1_S is associated with the evolution of five-membered ring from the M1H1_S, where O8-H10 group and H9 atom belonging to the water move towards the C1 atom and the terminal oxygen atom (O4) of the C1O3O4 group belonging to stabCI-OO, respectively. There are also significant changes in the length of the main bonds. In particular, the O4–H9 bond length (1.408 Å) in the corresponding transition (TSM12H1_S) complex decreases by 0.429 Å and O8–H9 bond (1.103 Å) increases by 0.121 Å compared with those in M1H1_S. The distance between C1 and O8 in the TSM12H1_S is 2.037 Å. Then, the α-hydroxy hydroperoxide M1H1_S can be formed according to the following scheme: both C1–O8 and O4–H9 distances in the TSM12H1_S continue to decrease, leading to O8–H9 bonds broken and the formation of both C1-O8 (1.402Å) and O4–H9 (0.971Å) bonds in the M3H1_S.

The M3H1_S has six different reaction pathways (b1H1_S-b6H1_S). In the reaction pathway b1H1_S, M3H1_s initially evolves via the transition state TSM31H1_S. Then, the O3–O4 bond (2.452 Å) of TSM31H1_S is broken and the formation of R2H1_S and O4H9 radicals occurs. The b1H1_S of reaction (2) may be considered as another possible source of atmospheric OH radicals.

In the reaction pathway b2H1_S/b3H1_S, M3H1_S evolves via the transition state TSM32H1_S/TSM33H1_S, yielding M5H1_S/M6H1_S. This process occurs according to the following scheme: the H5/H6 connected to C2 moves towards O4–H9 group, while H7 linked to C1 migrates to C2 instead of H5/H6. After both O4-H5/O4-H6 (0.967 Å/0.965 Å) and C2-H7 (1.093 Å/1.906 Å) bonds are formed, the formation of M5H1_S/M6H1_S is completed. The O3–O4 bond increases to 2.274 Å/2.268 Å in TSM32H1_S/TSM33H1_S, and elongates to 3.148 Å/2.923 Å in M5H1_S/M6H1_S. Finally, the O3–O4 bond in the M5H1_S/M6H1_S is broken, leading to the formation of limononic acid A/limononic acid B and water. The reaction pathway b4H1_S is similar to b2H1_S. H7 connected to C1 migrates to the O4–H9 group, forming O4–H7 bond. The M3H1_S transforms via the transition state TSM34H1_S, into M7H1_S. Finally, the limononic acid C and water are formed from M7H1_S. The reaction pathway b5H1_S is quite similar to b2H1_S. However, in contrast to the b2H1_S, H7 connected to C1 moves towards O3, while H10 bonded to O8 migrates to O4–H9 group. After O8–H10 bond is broken and both O3–H7 (0.989 Å) and O4-H10 (0.975 Å) bonds are formed, the formation M8H1_S is completed. Then, the O3-O4 bond in the M8H1_S gets broken, leading to the formation of limononic acid D and water. In the reaction pathway b6H1_S, M3H1_S evolves via the transition state TSM36H1_S, into M9H1_S. This process occurs according to the following scheme: H10 connected to O8 moves towards O3 and the distance of O8-H10 increases by 0.720 Å reaching 1.687 Å, while the C1–O3 bond is elongated by 1.980 Å reaching 3.411 Å in TSM36H1_S. TSM36H1_S then transforms into M9H1_S as the H10 connected to O8 continue to move towards O3 and the distance of O8-H10 gets increased to 1.839 Å, while the C1–O3 distance increases to 3.477 Å. After the O3–H10 bond (0.983 Å) is formed, the formation of M9H1_S is completed. Finally, the limonoaldehyde and H_2_O_2_ are formed from M9H1_S. The b6H1_S can also be considered as a possible source of atmospheric H_2_O_2_.

Scheme 3 and Figure 2 show that the reaction of stabCI-CH_3_-OO with H_2_O exhibits the same behavior as stabCI-OO + H_2_O reaction. At the first step, the reacting stabCI-CH_3_-OO and H_2_O evolve into a hydrogen-bond M1H2_S complex of seven-membered ring. The M1H2_S finally transforms via the transition state TSM11H2_S (reaction (1)) into OH and R1H2_S radicals (pathway (aH2_S)) and via the TSM12H2_S (reaction (2)) into OH radical, R2H2_S radical, H_2_O, R3H2_S, R4H2_S, limononaldehyde and H_2_O_2_ (pathway (b1H2_S–b3H2_S)).

Scheme 4 and Figure 3 also show that the reaction between stabCI*x*-OO and H_2_O exhibits the same pattern and occurs according to the same scheme as those for the reaction between stabCI-CH_3_-OO and H_2_O. The reaction between stabCI*x*-OO and H_2_O leads to the formation of a hydrogen-bonded M1H3_S complex with seven-membered ring. Finally, the M1H3_S finally evolves via the transition states TSM11H3_S (reaction (1)) and TSM12H3_S (reaction (2)) into OH radical, R1H3_S radical, R2H3_S radical, R3H3_S, R4H3_S, H_2_O, Keto-limonene and H_2_O_2_.

### 3.2. Thermochemical Analysis

Although the higher level CCSD(T)/6-31G(d) + CF method has been chosen as a principal computational method for the present study, in order to ensure the quality of the obtained results and to validate the obtained conclusions, reaction and activation energies for the reaction between stabCl-OO and H_2_O with the Zero-Point Correction (ZPE) included were also calculated using several other methods used to study the similar systems in the past. The results of the present study are given in Table 1. As it may be seen from Table 1, the reaction and activation energies calculated at different levels are in qualitative agreement in most of the cases studied (not exceed 10 kcal mol^−1^). Only in a few cases, such as the energy of TSM31H1_S relative to M3H1_S, the values predicted B3LYP/6-31G(d,p), MP2/6-31G(d), MP2/6-311++G(d,p) and CCSD(T)/6-31G(d) are quite different from those given by the CCSD(T)/6-31G(d) + CF with the differences of 23.75 kcal mol^−1^, 21.22 kcal mol^−1^, 27.14 kcal mol^−1^ and 2.53 kcal mol^−1^ respectively, presumably most accurate method used in the present study.

**Figure 4:**
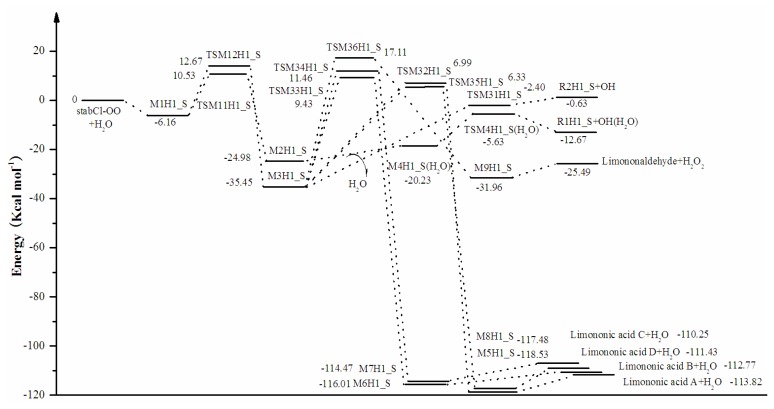
stabCI-OO + H_2_O reaction coordinates: relative energies of the stationary points located on the separate stabCI-OO and H_2_O ground-state potential energy surface. The energy values are given in kcal mol^−1^ and were calculated using CCSD(T)/6-31G(d) + CF//B3LYP/6-31G(d,p) level.

As seen from Table 1 and Figure 4, the most accurate and higher level CCSD(T)/6-31G(d) + CF method show that the initial hydrogen-bond complex M1H1_S is 6.16 kcal mol^−1^ more stable than the separate stabCl-OO and H_2_O. For the reaction of stabCI-OO with H_2_O, the formation of α-hydroxy hydroperoxide (reaction (2)) (−35.45 kcal mol^−1^) is more favorable than the formation of OH radicals with water-catalyzed H migration path (reaction (1)) (−24.98 kcal mol^−1^) (see Figure 4) The energies for the reaction pathways aH1_S and b1H1_S associated with the formation of OH radical are 12.67 kcal mol^−1^ and 0.63 kcal mol^−1^, respectively. Among the seven reaction pathways (aH1_S-b6H1_S), the pathways (aH1_S and b1H1_S) for the formation of OH radical are by 12.67 kcal mol^−1^ and 0.63 kcal mol^−1^ more stable than the separate stabCI-OO and H_2_O, the pathway (b6H1_S) for formation of limonoaldehyde and H_2_O_2_ is by 25.49 kcal mol^−1^ more stable than the separate stabCI-OO and H_2_O, and the four pathways (b2H1_S-b5H1_S) for formation of limononic acid and water are by 110.25-113.82 kcal mol^−1^ more stable than the separate stabCI-OO and H_2_O. The differences in the reaction energies are close and agree within 3.57 kcal mol^−1^ for all the four pathways (b2H1_S-b5H1_S).

With respect to the corresponding M1H1_S, the activation energy for the formation of OH radicals with water-catalyzed H migration path (reaction (1)) (16.69 kcal mol^−1^) is smaller than that for the formation of α-hydroxy hydroperoxide (reaction (2)) (18.83 kcal mol^−1^), indicating that the formation of OH radicals with water-catalyzed H migration path (reaction (1)) is slightly more favorable than the formation of α-hydroxy hydroperoxide (reaction (2)). Thus, with the reaction between our optimized stabCI-OO (syn stabCI-OO) and water, this conclusion is opposite to that obtained in the earlier theoretical study of the reaction between isoprene stabilized Criegee intermediates and H_2_O by Anglada [37]. Since in Anglada’s study [37], the energies for the reaction of water with all the eight isoprene stabilized Criegee intermediates have been calculated using G2(G2M-RCC5) method [38] with all stationary points optimized using the B3LYP/6-311+G(2d,2p) and neither comprehensive benchmarks for aforementioned method [38] nor a comparison between the prediction of the CCSD(T)/6-31G(d) + CF//B3LYP/6-31G(d,p) method and G2M-RCC5//B3LYP/6-311+G(2d,2p) methods are available at the present time, the activation energies between reaction (1) and reaction (2) for stabCI-OO and H_2_O obtained at the B3LYP/6-31G(d,p)//B3LYP/6-31G(d,p), MP2/6-31G(d)//B3LYP/6-31G(d,p), MP2/6-311++G(d,p)//B3LYP/6-31G(d,p) and CCSD(T)/6- 31G(d)//B3LYP/6-31G(d,p) levels of theory in the present study were used to validate our conclusion. The comparison, which indicates that the reaction (1) is more favorable than reaction (2) for stabCI-OO and H_2_O in all the cases studied (expect for the CCSD(T)/6-31G(d)//B3LYP/6-31G(d,p)) method) largely confirms our conclusion.

The activation energies of the six subsequent reaction pathways for the formation of α-hydroxy hydroperoxide (reaction (2)) are in the range of 33.04–52.55 kcal mol^−1^ with respect to corresponding M3H1_S, with the b1H1_S as the most favorable pathway of the activation energy of 33.04 kcal mol^−1^. The activation energies for the four pathways (b2H1_S-b5H1_S) are in the range of 41.78–46.91 kcal mol^−1^, and the differences between them are quite small (<3.578 kcal mol^−1^). Figure 4 illustrates the relative energies of the stationary points located on the singlet ground-state the separate stabCI-OO and H_2_O potential energy surface at the CCSD(T)/6-31G(d) + CF level of theory.

**Figure 5:**
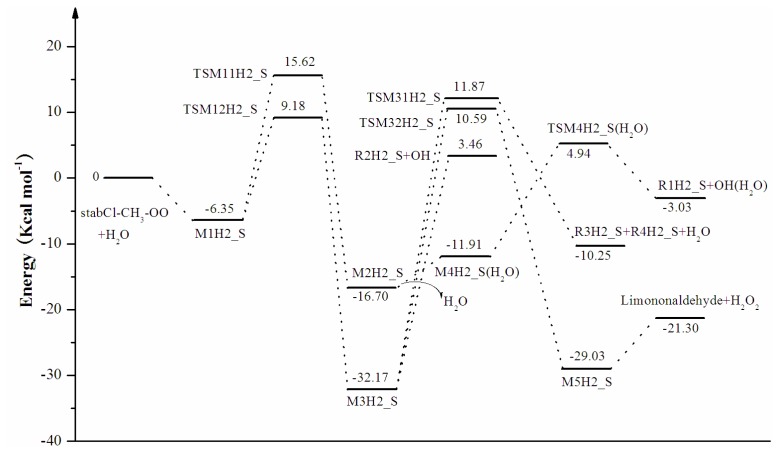
StabCI-CH_3_-OO + H_2_O reaction coordinates: relative energies of the stationary points located on the separate stabCI-CH_3_-OO and H_2_O ground-state potential energy surface. The energy values are given in kcal mol^−1^ and were calculated using CCSD(T)/6- 31G(d) + CF//B3LYP/6-31G(d,p) level.

Reaction and activation energies for the reaction between stabCI-CH_3_-OO and H_2_O with the Zero-Point Correction (ZPE) included were calculated at different levels of theory. The results of the calculations are presented in Table 2. As it may be seen from Table 2 and Figure 5, the higher level CCSD(T)/6-31G(d) + CF predicts that the initial hydrogen-bond complex M1H2_S is 6.35 kcal mol^−1^ more stable than the separate stabCI-CH_3_-OO and H_2_O. For the reaction of stabCI-CH_3_-OO with H_2_O, the formation of α-hydroxy hydroperoxide (reaction (2)) (−32.17 kcal mol^−1^) is more stable than the formation of OH radicals with the water-catalyzed H migration path (reaction (1)) (−16.70 kcal mol^−1^). (See Figure 5) In the case of the four following reaction pathways (aH2_S-b3H2_S), the reaction energies with the respect to corresponding to the separated stabCI-CH_3_-OO and H_2_O, are −3.03 kcal mol^−1^, −3.46 kcal mol^−1^, −10.25 kcal mol^−1^ and −21.30 kcal mol^−1^, respectively. The activation energies for the formation of OH radicals from M1H2_S via water-catalyzed H migration path (reaction (1)) (21.98 kcal mol^−1^) is larger than those for the formation of α-hydroxy hydroperoxide (reaction (2)) (15.53 kcal mol^−1^). This indicates that the formation of α-hydroxy hydroperoxide (reaction (2)) is more favorable than the formation of OH radicals with water-catalyzed H migration path (reaction (1)). The differences in the activation energies between reaction (1) and reaction (2) for stabCI-CH_3_-OO and H_2_O obtained at the B3LYP/6-31G(d,p)//B3LYP/6-31G(d,p), MP2/6-31G(d)//B3LYP/6-31G(d,p), MP2/6-311++G(d,p)//B3LYP/6-31G(d,p) and CCSD(T)/6- 31G(d)//B3LYP/6-31G(d,p) levels of theory also indicate that the reaction (2) is more favorable than reaction (1) for stabCI-OO and H_2_O. This conclusion is in agreement with the previous theoretical study of the reaction between isoprene stabilized Criegee intermediates and H_2_O by Anglada [37].

The activation energies of the three subsequent reaction pathways for the formation of α-hydroxy hydroperoxide (reaction (2)) are in the range of 35.63–44.04 kcal mol^−1^ with respect to corresponding M3H2_S, with the b1H2_S as the most favorable pathway with the activation energy of 35.63 kcal mol^−1^. Figure 5 illustrates the relative energies of the stationary points located on the singlet ground-state the separate stabCI-CH_3_-OO and H_2_O potential energy surface at the CCSD(T)/6-31G(d) + CF level of theory.

**Figure 6:**
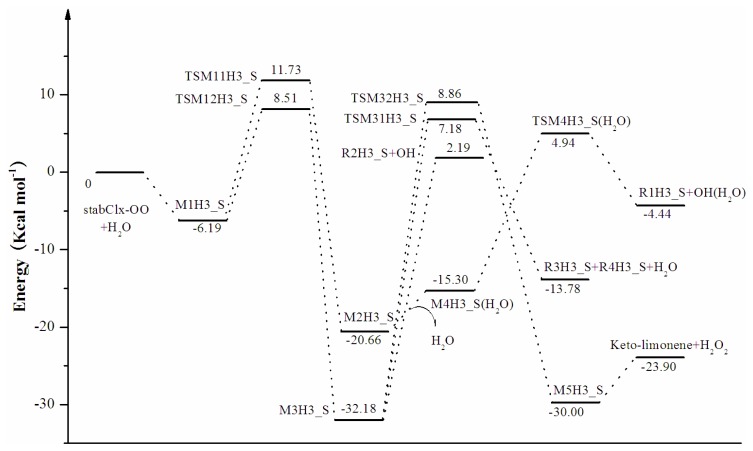
stabCI*x*-OO + H_2_O reaction coordinates: relative energies of the stationary points located on the separate stabCI*x*-OO and H_2_O ground-state potential energy surface. The energy values are given in kcal mol^−1^ and are calculated using CCSD(T)/6-31G(d) + CF//B3LYP/6-31G(d,p) level.

Reaction and activation energies for the reaction between stabCI*x*-OO and H_2_O with the Zero-Point Correction (ZPE) included calculated at different levels of theory are shown in Table 3. Table 3 and Figure 6 demonstrate that the values of reaction and activation for the reaction between stabCI*x*-OO and H_2_O correlate with those of reaction and activation energies for the reaction of stabCI-CH_3_-OO and H_2_O. The formation of α-hydroxy hydroperoxide (reaction (2)) is more favorable than the formation of OH radicals with water-catalyzed H migration path or reaction (1). The differences in the activation energies between reaction (1) and reaction (2) for stabCI*x*-OO and H_2_O obtained at the B3LYP/6-31G(d,p)//B3LYP/6-31G(d,p), MP2/6-31G(d)//B3LYP/6-31G(d,p), MP2/6-311++ G(d,p)//B3LYP/6-31G(d,p) and CCSD(T)/6-31G(d)//B3LYP/6-31G(d,p) levels of theory (4.06 kcal mol^−1^, 0.12 kcal mol^−1^, -0.73 kcal mol^−1^ and 7.16 kcal mol^−1^, respectively) also indicate (with exception for MP2/6-311++G(d,p)//B3LYP/6-31G(d,p) method) that the reaction (2) is likely more favorable than reaction (1) for stabCI*x*-OO and H_2_O. These findings are consistent with the previous theoretical study of the reaction between isoprene stabilized Criegee intermediates and H_2_O by Anglada [37]. Figure 6 illustrates the relative energies of the stationary points located on the singlet ground-state the separate stabCI*x*-OO and H_2_O potential energy surface at the CCSD(T)/6-31G(d) + CF level of theory.

As it may be seen from Tables 1–3, we can conclude that the energy of the initial hydrogen-bond complex (M1H1_S, M1H2_S, M1H3_S) are 6.16 kcal mol^−1^, 6.35 kcal mol^−1^ and 6.19 kcal mol^−1^ more stable than the corresponding separate stabilized Criegee intermediates (stabCl-OO, stabCI-CH_3_-OO, stabCI*x*-OO) and H_2_O at the CCSD(T)/6-31G(d) + CF level of theory. For the formation of OH radicals via the water-catalyzed H migration path (reaction (1)), the activation energies with the respect to hydrogen-bond complex (M1H1_S, M1H2_S, M1H3_S) are 16.68 kcal mol^−1^, 21.98 kcal mol^−1^, and 17.92 kcal mol^−1^, respectively, with the reaction (1) for the reaction of stabCl-OO and H_2_O as the most favorable pathway (16.68 kcal mol^−1^). For the formation of α-hydroxy hydroperoxide (reaction (2)), the activation energies are in the range of 14.70–18.83 kcal mol^−1^ with respect to corresponding hydrogen-bond complex (M1H1_S, M1H2_S, M1H3_S), with the reaction (2) for stabCI*x*-OO + H_2_O reaction as the most favorable pathway (14.70 kcal mol^−1^).

## 4. Conclusions

In the present study, several important aspects of the gas-phase reaction of the stabilized Criegee intermediates and H_2_O have been investigated.

The present study leads us to the following conclusions:

- The reaction between the stabilized Criegee intermediates (stabCI-OO, stabCI-CH_3_-OO and stabCI*x*-OO) and H_2_O is the three-step reaction. At the first stage, the formation of a hydrogen-bonded complex occurs. At the second stage, the reaction can proceed via the following two reaction pathways: (1) formation of OH radicals with water-catalyzed H migration; and (2) formation of α-hydroxy hydroperoxide. The formation of α-hydroxy hydroperoxide (reaction (2)), the reaction of stabCI-OO and H_2_O occurs via six different degradation pathways, while the reaction of stabCI-CH_3_-OO and stabCI*x*-OO with H_2_O occurs via three different reaction pathways.
- The formation of OH radicals with water-catalyzed H migration path (aH1_S, aH2_S and aH3_S) and formation of α-hydroxy hydroperoxide (reaction pathways b1H1_S, b1H2_S and b1H3_S) may be considered as possible sources of OH radicals in the Earth’s atmosphere. The formation of α-hydroxy hydroperoxide (reaction pathways b6H1_S, b3H2_S and b3H3_S) can be considered as possible sources of atmospheric H_2_O_2_.
- The CCSD(T)/6-31G(d) + CF activation energies are in the range of 14.70–21.98 kcal mol^−1^ with respect to corresponding hydrogen-bond complexes (M1H1_S, M1H2_S, M1H3_S) between stabilized Criegee intermediates and H_2_O. The formation of α-hydroxy hydroperoxide (reaction (2)) for the reaction of stabCI*x*-OO and H_2_O is the most favorable pathway with the activation energy of 14.70 kcal mol^−1^.
- For the reaction of stabCI-OO and H_2_O, with the reaction between our optimized stabCI-OO (syn stabCI-OO) and water, the formation of OH radicals with water-catalyzed H migration path or reaction (1) is more favorable than the formation of α-hydroxy hydroperoxide or reaction (2). This conclusion is opposite to that obtained in the previous theoretical study of the reaction between isoprene stabilized Criegee intermediates and H_2_O by Anglada. For the reaction of stabCI-CH_3_-OO and stabCI*x*-OO with H_2_O, the formation of α-hydroxy hydroperoxide (reaction (2)) is more favorable than the formation of OH radicals with water-catalyzed H migration path or reaction (1). This conclusion is consistent with results of the previous theoretical study of the reaction between isoprene stabilized Criegee intermediates and H_2_O by Anglada.

## Figures and Tables

**Table 1:** Reaction and activation energies (*E*) with Zero-Point Correction (ZPE) included (kcal mol^−1^)) for the reaction between stabCl-OO and H_2_O a at different levels of theory.

Compound	Relative to	*E*(B3LYP/6-31G(d,p))	*E*(MP2/6-31G(d))	*E*(MP2/6-311++G(d,p))	*E*(CCSD(T)/6-31G(d))	*E*(CCSD(T)/6-31G(d) + CF)
stabCl-OO + H_2_O		0.00	0.00	0.00	0.00	0.00
M1H1_S	stabCl-OO + H_2_O	−8.65	−7.27	−7.54	−7.54	−6.16
TSM11H1_S	M1H1_S	13.27	11.44	8.52	18.51	16.68
M2H1_S	TSM11H1_S	−34.17	−34.57	−26.94	−35.09	−35.50
M4H1_S + H_2_O	M2H1_S	8.80	5.64	8.50	7.91	4.74
TSM4H1_S	M4H1_S	24.29	18.38	29.60	20.51	14.60
R1H1_S+OH	TSM4H1_S	−10.64	-8.29	−17.17	−9.39	−7.04
TSM12H1_S	M1H1_S	14.96	17.01	13.62	16.78	18.83
M3H1_S	TSM12H1_S	−50.15	−50.35	−41.65	−47.92	−48.12
TSM31H1_S	M3H1_S	56.79	54.26	60.18	35.57	33.04
R2H1_S+OH	TSM31H1_S	−12.35	−12.64	−22.88	2.06	1.78
TSM32H1_S	M3H1_S	40.23	32.82	46.83	49.85	42.44
M5H1_S	TSM32H1_S	−121.60	−120.87	−124.12	−126.25	−125.52
Limononic acid A + H_2_O	M5H1_S	7.41	5.01	6.31	7.12	4.71
TSM33H1_S	M3H1_S	44.84	35.62	51.29	54.09	44.87
M6H1_S	TSM33H1_S	−121.88	−120.57	−126.09	−126.76	−125.44
Limononic acid B + H_2_O	M6H1_S	5.84	3.56	5.60	5.53	3.24
TSM34H1_S	M3H1_S	53.34	48.18	51.67	52.07	46.91
M7H1_S	TSM34H1_S	−130.73	−131.72	−126.97	−124.94	−125.93
Limononic acid C + H_2_O	M7H1_S	8.06	4.72	7.79	7.56	4.22
TSM35H1_S	M3H1_S	44.65	42.72	34.16	43.70	41.78
M8H1_S	TSM35H1_S	−125.34	−129.75	−111.65	−119.40	−123.81
Limononic acid D + H_2_O	M8H1_S	8.44	6.46	7.78	8.03	6.05
TSM36H1_S	M3H1_S	54.94	55.16	49.52	52.33	52.55
M9H1_S	TSM36H1_S	−51.97	−51.66	−47.33	−49.38	−49.07
Limononaldehyd e + H_2_O_2_	M9H1_S	8.58	6.79	8.12	8.26	6.47

a Optimized geometries, vibrational frequencies and ZPE obtained at the B3LYP/6-31G(d,p) level.

**Table 2:** Reaction and activation energies (*E*) with Zero-Point Correction (ZPE) included (kcal mol^−1^) for the reaction between stabCl-CH_3_-OO and H_2_O a at different levels of theory.

Compound	Relative to	*E*(B3LYP/6-31G(d,p))	*EG*(MP2/6-31G(d))	*E*(MP2/6-311++G(d,p))	*E*(CCSD(T)/6-31G(d))	*E*(CCSD(T)/6-31G(d) + CF)
stabCl-CH_3_-OO + H_2_O		0.00	0.00	0.00	0.00	0.00
M1H2_S	stabCl-CH_3_- OO + H_2_O	−11.15	−7.94	−10.45	−9.57	−6.35
TSM11H2_S	M1H2_S	19.60	16.93	14.68	24.64	21.98
M2H2_S	TSM11	−30.19	−31.48	−22.41	−31.04	−32.32
M4H2_S + H_2_O	M2H2_S	8.74	5.66	8.71	7.86	4.79
TSM4H2_S	M4H2_S	29.25	25.80	41.29	20.30	16.86
R1H2_S+OH	TSM4H2_S	−17.41	−15.58	−28.27	−9.81	−7.98
TSM12H2_S	M1H2_S	11.72	13.61	11.25	13.64	15.53
M3H2_S	TSM12H2_S	−43.36	−43.58	−34.17	−41.13	−41.35
R2H2_S + OH	M3H2_S	46.53	43.33	38.15	38.84	35.63
TSM31H2_S	M3H2_S	45.47	43.40	37.90	46.11	44.04
R3H2_S + R4H2_S + H_2_O	TSM31H2_S	−20.33	−22.99	−21.49	−19.45	−22.11
TSM32H2_S	M3H2_S	44.87	43.12	36.08	44.51	42.76
M5H2_S	TSM32H2_S	−41.41	−39.53	−35.87	−41.49	−39.62
Limononaldehyde + H_2_O_2_	M5H2_S	9.16	8.17	8.14	8.72	7.73

a Optimized geometries, vibrational frequencies and ZPE obtained at the B3LYP/6-31G(d,p) level.

**Table 3:** Reaction and activation energies (*E*) with Zero-Point Correction (ZPE) included (kcal mol^−1^) for the reaction between stabCl*x*-OO and H_2_O a at different levels of theory.

Compound	Relative to	*E*(B3LYP/6-31G(d,p))	*E*(MP2/6-31G(d))	*E*(MP2/6-311++G(d,p))	*E*(CCSD(T)/6-31G(d))	*E*(CCSD(T)/6-31G(d) + CF)
stabClx-OO + H_2_O		0.00	0.00	0.00	0.00	0.00
M1H3_S	stabClx-OO + H_2_O	−10.79	−7.67	−10.22	−9.32	−6.19
TSM11H3_S	M1H3_S	15.56	12.93	11.36	20.55	17.92
M2H3_S	TSM11H3_S	−30.54	−31.66	−22.01	−31.27	−32.39
M4H3_S + H_2_O	M2H3_S	9.31	6.32	8.79	8.35	5.36
TSM4H3_S	M4H3_S	28.12	24.23	34.67	24.14	20.24
R1H3_S+OH	TSM4H3_S	−12.80	−11.39	−18.84	−10.79	−9.38
TSM12H3_S	M1H3_S	11.50	12.81	12.09	13.39	14.70
M3H3_S	TSM12H3_S	−42.89	−42.86	−33.40	−40.72	−40.69
R2H3_S + OH	M3H3_S	45.32	42.42	35.58	37.28	34.38
TSM31H3_S	M3H3_S	41.90	38.07	34.61	43.19	39.36
R3H3_S + R4H3_S + H_2_O	TSM31H3_S	−19.76	−20.40	−24.55	−20.32	−20.95
TSM32H3_S	M3H3_S	42.47	41.24	34.04	42.27	41.04
M5H3_S	TSM32H3_S	−40.78	−38.49	−36.13	−41.14	−38.86
Keto-limonene + H_2_O_2_	M5H3_S	8.20	6.41	7.99	7.88	6.10

a Optimized geometries, vibrational frequencies and ZPE obtained at the B3LYP/6-31G(d,p) level.

## References

[1] Hatakeyama S., Akimoto H. (1994). Reactions of Criegee intermediates in the gas phase. Res. Chem. Intermed.

[2] Ryzhkov A.B., Ariya P.A. (2003). A theoretical study of the reactions of carbonyl oxide with water in atmosphere: The role of water dimer. Chem. Phys. Lett.

[3] Criegee R. (1975). Mechanism of ozonolysis. Angew. Chem. Int. Ed. Engl.

[4] Neeb P., Horie O., Moortgat G.K. (1995). The nature of the transitory product in the gas-phase ozonolysis of ethane. Chem. Phys. Lett.

[5] Hatakeyama S., Bandow H., Okuda M., Akimoto H. (1981). Reactions of peroxymethylene and methylene (1A1) with water in the gas phase. J. Phys. Chem.

[6] Crehuet R., Anglada J.M., Bofill J.M. (2001). Tropospheric formation of hydroxymethyl hydroperoxide, formic acid, H_2_O_2_, and OH from carbonyl oxide in the presence of water vapor: A theoretical study of the reaction mechanism. Chem. Eur. J.

[7] Jenkin M.E., Saunders S.M., Pilling M.J. (1997). The tropospheric degradation of volatile organic compounds: A protocol for mechanism development. Atmos. Environ.

[8] Gäb S., Hellpointner E., Turner W.V., Korte F. (1985). Hydroxymethyl hydroperoxide and bis(hydroxymethy1) peroxide from gas-phase ozonolysis of naturally occurring alkenes. Nature.

[9] Barnes I., Bastian V., Becker K.H., Zhu T. (1990). Kinetics and products of the reactions of nitrate radical with monoalkenes, dialkenes, and monoterpenes. J. Phys.Chem.

[10] Becke A D. (1993). Density-functional thermochemistry. III. The role of exact exchange. J. Phys. Chem..

[11] Neeb P., Sauer F., Horie O., Moortgat G.K. (1997). Formation of hydroxymethyl hydroperoxide and formic acid in alkene ozonolysis in the presence of water vapour. Atmos. Environ.

[12] Hasson A.S., Orzechowska G., Paulson S.E. (2001). Production of stabilized Criegee intermediates and peroxides in the gas phase ozonolysis of alkenes 1. ethene, *trans*-2-butene, and 2,3-dimethyl-2- butene. J. Geophys. Res.

[13] Hasson A.S., Ho A.W., Kuwata K.T., Paulson S.E. (2001). Production of stabilized Criegee intermediates and peroxides in the gas phase ozonolysis of alkenes 2. asymmetric and biogenic alkenes. J. Geophys. Res.

[14] Sauer F., Schafer C., Neeb P., Horie O., Moortgat G.K. (1999). Formation of hydrogen peroxide in the ozonolysis of isoprene and simple alkenes under humid conditions. Atmos. Environ.

[15] Hewitt C.N., Kok G.L. (1991). Formation and occurrence of organic hydroperoxides in the troposphere: Laboratory and field observations. J. Atmos. Chem.

[16] Simonaitis R., Olsyna K.J., Meagher J.F. (1991). Production of hydrogen peroxide and organic peroxides in the gas phase reactions of ozone with natural alkenes. Geophys. Res. Lett.

[17] Martinez R.I., Herron J.T. (1981). Gas-phase reaction of SO_2_ with a Criegee intermediate in the presence of water vapor. J. Environ. Sci. Health A.

[18] Wolff S., Boddenberg A., Thamm J., Turner W.V., Gäb S. (1997). Gas-phase ozonolysis of ethene in the presence of carbonyl-oxide scavengers. Atmos. Environ.

[19] Winterhalter R., Neeb P., Grossmann D., Kolloff A., Horie O., Moortgat G.K. (2000). Products and mechanism of the gas phase reaction of ozone with β-pinene. J. Atmos. Chem.

[20] Puxbaum H., Rosenberg C., Gregori M., Lanzerstorfer C., Ober E., Winiwarter W. (1988). Atmospheric concentrations of formic and acetic acid and related compounds in eastern and northern Austria. Atmos. Environ.

[21] Hellpointner E., Gäb S. (1989). Detection of Methyl, Hydroxymethyl and hydroxyethyl hydroperoxides in air and precipitation. Nature.

[22] Grosjean D. (1989). Organic acids in southern California air: Ambient concentrations, mobile source emissions, *in situ* formation and removal processes. Environ. Sci. Technol.

[23] Sanhueza E., Santana M., Hermoso M. (1992). Gas- and aqueous-phase formic and acetic acids at a tropical cloud forest site. Atmos. Environ.

[24] Lawrence J.E., Koutrakis P. (1994). Measurement of atmospheric formic and acetic acids: Methods evaluation and results from field studies. Environ. Sci. Technol.

[25] Granby K., Christensen C.S., Lohse C. (1997). Urban and semi-rural observations of carboxylic acids and carbonyls. Atmos. Environ.

[26] Marklund S. (1973). Mechanisms of the irreversible inactivation of horseradish peroxidase caused by hydroxymethyl hydroperoxide. Arch. Biochem. Biophys.

[27] Möller D. (1989). The possible role of H_2_O_2_ in new-type forest decline. Atmos. Environ.

[28] Hewitt C.N., Kok G.L., Fall R. (1990). Hydroxyperoxides exposed to ozone mediate air pollution damage to alkene emitters. Nature.

[29] Calvert J.G., Lazrus A., Kok G.L., Heikes B.G., Walega J.G., Lind J., Cantrell C.A. (1985). Chemical mechanisms of acid generation in the troposphere. Nature.

[30] Niki H., Maker P.D., Savage C.M., Breitenbach L.P., Hurley M.D. (1987). FTIR spectroscopic study of the mechanism for the gas-phase reaction between ozone and tetramethylethylene. J. Phys. Chem.

[31] Jonsson Å., Hallquist M., Ljungström E. (2008). Influence of OH scavenger on the water effect on secondary organic aerosol formation from ozonolysisof limonene, Δ^3^-carene, and α-pinene. Environ. Sci. Technol..

[32] Leungsakul S., Jaoui M., Kamens R.M. (2005). Kinetic Mechanism for predicting secondary organic aerosol formation from the reaction of D-limonene with ozone. Environ. Sci. Technol.

[33] Dowideit P., von Sonntag C. (1998). Reaction of ozone with ethene and its methyl- and chlorine-substituted derivatives in aqueous solution. Environ. Sci. Technol.

[34] Anglada J.M., González J., Torrent-Sucarrat M. (2011). Effects of the substituents on the reactivity of carbonyl oxides. A theoretical study on the reaction of substituted carbonyl oxides with water. Phys. Chem. Chem. Phys.

[35] Aplincourt P., Ruiz-López M.F. (2000). Theoretical study of formic acid anhydride formation from carbonyl oxide in the atmosphere. J. Phys. Chem. A.

[36] Anglada J.M., Aplincourt P., Bofill J.M., Cremer D. (2002). Atmospheric formation of OH radicals and H_2_O_2_ from alkene ozonolysis under humid conditions. Chemphyschem.

[37] Aplincourt P., Anglada J.M. (2003). Theoretical studies on isoprene ozonolysis under tropospheric conditions. 1. Reaction of substituted carbonyl oxides with water. J. Phys. Chem. A.

[38] Mebel A.M., Morokuma K., Lin M.C. (1995). Modification of the Gaussian-2 theoretical model: The use of coupled cluster energies, densityfunctional geometries, and frequencies. J. Chem. Phys.

[39] Guenther A., Geron C., Pierce T., Lamb B., Harley P., Fall R. (2000). Natural emissions of non-methane volatile organic compounds, carbon monoxide, and oxides of nitrogen from North America. Atmos. Environ.

[40] Ramírez-Ramírez V.M., Nebot-Gil I. (2005). Theoretical study of the OH addition to the endocyclic and exocyclic double bonds of the D-limonene. Chem. Phys. Lett.

[41] Jiang L., Xu Y.S., Ding A.Z. (2010). Reaction of stabilized Criegee intermediates from ozonolysis of limonene with sulfur dioxide: *Ab initio* and DFT study. 2010. J. Phys. Chem. A.

[42] Wang Y.D. (2008). Theory Study on Mechanism of O_3_-Initiated Atmospheric Photooxidation of Terpenes. Master’s Dissertation.

[43] Leungsakul S., Jeffries H.E., Kamens R.M. (2005). A kinetic mechanism for predicting secondary aerosol formation from the reactions of D-limonene in the presence of oxides of nitrogen and natural sunlight. Atmos. Environ.

[44] Neeb P., Horie O., Moortgat G.K. (1998). The ethene-ozone reaction in the gas phase. J. Phys. Chem. A.

[45] Herron J.T., Huie E. (1977). Stopped-flow studies of the mechanisms of ozone-alkene reactions in the gas phase. Ethylene. J. Am. Chem. Soc.

[46] Martinez R.I., Herron J.T. (1988). Stopped-flow studies of the mechanisms of ozone-alkene reactions in the gas phase: Trans-2-butene. J. Phys. Chem.

[47] Gutbrod R., Kraka E., Schindler R.N., Cremer D. (1997). Kinetic and theoretical investigation of the gas-phase ozonolysis of isoprene: Carbonyl oxides as an important source for OH radicals in the atmosphere. J. Am. Chem. Soc.

[48] Anglada J.M., Bofill J.M., Olivella S., Sole A. (1998). Theoretical investigation of the low-lying electronic states of dioxirane: Ring opening to dioxymethane and dissociation into CO_2_ and H_2_. J. Phys. Chem. A.

[49] Chen B.Z., Anglada J.M., Huang M.B., Kong F. (2002). The reaction of CH_2_ (X_3_B1) with O_2_ (X_3_ ): A theoretical CASSCF/CASPT2 investigation. J. Phys. Chem. A.

[50] Paulson S.E., Chung M.Y., Hasson A.S. (1999). OH radical formation from the gas-phase reaction of ozone with terminal alkenes and the relationship between structure and mechanism. J. Phys. Chem. A.

[51] Aplincourt P., Ruiz-López M.F. (2000). Theoretical investigation of reaction mechanisms for carboxylic acid formation in the atmosphere. J. Am. Chem. Soc.

[52] Frisch M.J., Trucks G.W., Schlegel H.B., Scuseria G.E., Robb M.A., Cheeseman J.R., Montgomery J.A., Vreven T., Kudin K.N., Burant J.C. Gaussian 03, Revision E.01.

[53] Hariharan P.C., Pople J.A. (1973). The influence of polarization functions on molecular orbital hydrogenation energies. Theor. Chim. Acta.

[54] Gonzalez C., Schlegel H.B. (1989). An improved algorithm for reaction path following. J. Chem. Phys.

[55] Baptista L., Pfeifer R., da Silva E.C., Arbilla G. (2011). Kinetics and thermodynamics of limonene ozonolysis. J. Phys. Chem. A.

[56] Szabo A., Ostlund N.S. Modern Quantum Chemistry: Introduction to Advanced Electronic Structure Theory.

[57] Lei W., Derecskei-Kovacs A., Zhang R. (2000). *Ab initio* study of OH addition reaction to isoprene. J. Chem. Phys.

[58] Suh I., Lei W., Zhang R. (2001). Experimental and theoretical studies of isoprene reaction with NO_3_. J. Phys. Chem. A.

[59] Zhang D., Zhang R. (2002). Mechanism of OH formation from ozonolysis of isoprene: A quantum-chemical study. J. Am. Chem. Soc.

[60] Jiang L., Wang W., Xu Y.S. (2009). Theoretical investigation of the NO_3_ radical addition to double bonds of limonene. Int. J. Mol. Sci.

[61] Jiang L., Wang W., Xu Y.S. (2010). *Ab initio* investigation of O_3_ addition to double bonds of limonene. Chem. Phys.

[62] Gutbrod R., Schindler R.N., Kraka E., Cremer D. (1996). Formation of OH radicals in the gas phase ozonolysis of alkenes: The unexpected role of carbonyl oxides. Chem. Phys. Lett.

[63] Olzmann M., Kraka E., Cremer D., Gutbrod R., Andersson S. (1997). Energetics, kinetics, and product distributions of the reactions of ozone with ethene and 2,3-dimethyl-2-butene. J. Phys. Chem. A.

[64] Cremer D., Kraka E., Szalay P.G. (1998). Decomposition modes of dioxirane, methyldioxirane and dimethyldioxirane—A CCSD(T), MR-AQCC and DFT investigation. Chem. Phys. Lett.

[65] Xu F., Wang H., Zhang Q.Z., Zhang R.X., Qu X.H., Wang W.X. (2010). Kinetic properties for the complete series reactions of chlorophenols with OH radicals—Relevance for dioxin formation. Environ. Sci. Technol.

[66] Sun X.Y., He M.X., Zhang Q.Z., Wang W.X., Jalbout A.F. (2008). Quantum chemical study on the atmospheric photooxidation of Methyl Vinyl Ether (MVE). J. Mol. Struc.

[67] Drozd G.T., Kroll J., Donahue N.M. (2011). 2,3-Dimethyl-2-butene (TME) ozonolysis: Pressure dependence of stabilized criegee intermediates and evidence of stabilized vinyl hydroperoxides. J. Phys. Chem. A.

[68] Kurtén T., Donahue N.M. (2012). MRCISD studies of the dissociation of vinylhydroperoxide, CH_2_CHOOH: There is a saddle point. J. Phys. Chem. A.

